# Topsoil and Vegetation Dynamics 14 Years after *Eucalyptus grandis* Removal in Eastern Cape Province of South Africa

**DOI:** 10.3390/plants12173047

**Published:** 2023-08-24

**Authors:** Kuhle Mthethwa, Sheunesu Ruwanza

**Affiliations:** 1 Department of Environmental Science, Rhodes University, Makhanda 6140, South Africa; kuhlemthethwa.h@gmail.com; 2 Department of Environmental Science, Centre of Excellence for Invasion Biology, Rhodes University, Makhanda 6140, South Africa

**Keywords:** biological invasion, invasive alien plants, ecological restoration, plant–soil recovery, follow-up clearing

## Abstract

A great deal of effort has been made to clear invasive alien plants in South Africa, yet it remains unclear if the clearing efforts are yielding positive soil and vegetation recovery trajectories. A few short-term studies have been conducted to monitor soil and vegetation recovery after alien plant removal in South Africa, but convincing, long-term monitoring studies are scarce yet needed. We investigated topsoil and vegetation recovery following *Eucalyptus grandis* removal 14 years ago by Working for Water in Makhanda, Eastern Cape province of South Africa. The detailed topsoil and vegetation surveys were conducted on forty 10 m × 10 m plots that were in paired cleared and natural sites. The results show no significant differences for the measured soil pH, total N, total C, K, Ca, and Na between the cleared and natural sites, an indication that the two sites are becoming similar. Similarly, the gravimetric soil moisture content shows no significant differences between the two sites, although monthly variations are observed. The topsoils in the cleared sites are hydrophobic as compared to those in the natural sites, which are wettable. We observed no significant vegetation diversity differences between the two sites, with native woody species, such as *Crassula pellucida* and *Helichrysum petiolare,* frequently occurring in the cleared sites. We recorded low reinvasion by *E. grandis* and other secondary invaders like *Acacia mearnsii* and *Rubus cuneifolius* in the cleared sites. Based on these results, we conclude that 14 years after *E. grandis* clearing, both topsoil and vegetation recovery are following a positive trajectory towards the natural sites. However, both reinvasion and secondary invasion have the potential to slow down soil and native vegetation recovery. Recommendations such as timeous follow-up clearing and incorporating restoration monitoring in the WfW clearing programme are discussed.

## 1. Introduction

Invasion by invasive alien plants is a major threat to South Africa’s socio-economic and ecological environment [1]. For example, O’Connor and van Wilgen [2] reported that an invasion of South Africa’s rangelands by invasive alien plants, such as *Acacia*, *Eucalyptus*, *Opuntia*, and *Prosopis* species, can negatively affect livestock grazing and subsequently reduce livestock production by an estimated ZAR 340 million per year. In the Western and Eastern Cape provinces of South Africa, invasion by some of the above-mentioned alien plant species has been shown to negatively affect water resources and the country’s natural vegetation [3]. From a social standpoint, invasion by invasive alien plants, such as *A. dealbata*, increases local people’s vulnerability through a reduction in crop yields and grazing lands [4]. In urban areas, invasive alien plants contribute towards the homogenisation of city habitats, clogging water canals resulting in flooding, soil erosion, and disrupting ecosystem services, such as water infiltration [5,6]. These negative socio-economic and ecological effects caused by invasive alien plants need to be reversed, thus the need for restoration after alien plant control and management [7].

In South Africa, the control and management of invasive alien plants have been performed by the Working for Water (WfW) programme since its inception in 1995 [8]. The WfW programme is a poverty alleviation initiative that champions the control of invasive alien plants to protect and maximise water resources in the country [9]. The programme assumes that clearing invasive alien plants will result in the passive restoration of ecosystems to their original condition [9]. To date, an estimated ZAR 15 billion has been used to control invasive alien plants at a rate of approximately 200,000 condensed ha per year [10]. Generally, the programme is regarded as a success, although several challenges have been reported, e.g., having dual objectives, which negatively affect budget prioritisation, inefficiencies associated with having multiple country-wide projects, lack of restoration goals post alien plant clearing, and funding limitations [9,10]. Besides the above-mentioned challenges, little is known regarding how cleared areas recover post alien plant clearing by WfW [11]. Although few studies have been conducted in South Africa to assess ecosystem recovery after invasive alien plant removal by WfW, some studies have shown little vegetation recovery due to secondary invasion and a lack of native species soil seed banks [11,12], whereas others have reported a positive vegetation recovery trajectory [11,13,14]. Most of the above-mentioned studies were short-term monitoring studies that were conducted less than five years after the initial alien plant clearing, thus failing to give a clear picture of the restoration trajectory. Therefore, there is an urgent need to conduct long-term restoration studies after invasive alien plant clearing to understand recovery trajectories and develop effective restoration guidelines across varied contexts [11,15,16,17].

Long-term restoration monitoring after alien plant clearing is needed if the WfW clearing initiative is to yield positive ecosystem recovery outcomes. Although short-term restoration studies after alien plant clearing are important, they fail to investigate the recovery trajectories over time and justify the restoration funding since some goals might not have been achieved [18]. Therefore, this means that monitoring interventions cannot be implemented or tracked over time [18]. In contrast, long-term monitoring of invasive alien plant-cleared sites has the potential to assess the recovery trajectory over a long period as well as assess changes over varied environmental and climatic events. In addition, long-term monitoring can improve restoration effectiveness by implementing interventions that will also be monitored. In addition, it allows for restoration decisions to be made based on generated long-term data [19]. Therefore, long-term ecological restoration monitoring following alien plant removal is essential for investigating ecosystem recovery trajectories, thus providing important information that can be used to inform future restoration initiatives [20].

Most ecological restoration studies following alien plant removal have monitored the recovery of vegetation by measuring species abundance, composition, and diversity [11] but neglected soil monitoring. For example, Ruwanza et al. [11,13] conducted both short- and medium-term vegetation recovery monitoring following *E. camaldulensis* removal in the Western Cape province of South Africa and reported that the vegetation composition is dominated by grasses and herbs during the early stages of restoration but changes over time as shrubs and trees start to recruit in the cleared sites. Fill et al. [15] reported the dominance of native riparian shrubs following the removal of invasive alien plants along the riparian zones of the Rondegat River in South Africa; however, a high diversity of alien grasses was also reported. Very few restoration studies have monitored changes in the soil properties after alien plant removal [14,21], yet soil recovery has the potential to influence plant composition through soil–plant interactions [22,23]. Both Ndou and Ruwanza [14] and Kerr and Ruwanza [24] reported mixed results (both increases and decreases in soil nutrients depending on the measured property) in soil recovery following *Acacia* and *Eucalyptus* (respectively) removal in the Eastern Cape province of South Africa. Nsikani et al. [25] reported that the soil nitrogen levels remain high in soils after *A. saligna* removal, an indication that soil legacy persistence has the potential to negatively affect vegetation recovery through promoting the growth of weedy secondary invaders. Methodologically, the bulk of the above-mentioned studies [14,24,25] on soil recovery after alien plant removal have assessed topsoil because it is the main source of soil nutrients and organic matter that is used by recruiting vegetation. Also, topsoil is assessed in restoration studies because it is a major repository of soil microbes that are known to influence the decomposition of plant debris, thus shaping both the above and below-ground vegetation recruitment trajectory. Although assessment of both the top and below-ground soil properties can yield more accurate results, the assessment of topsoil (which was performed in this study) can provide valuable information that can be used to assess the ecosystem recovery after alien plant removal. Our emphasis on topsoil measurements is centred on their role in influencing vegetation recovery after alien plant clearing, i.e., topsoil supply recruiting plants with valuable nutrients.

This study is motivated by the need for long-term monitoring of soil and vegetation recovery to gauge the effectiveness of alien plant clearing by WfW. To our knowledge, few long-term ecological restoration monitoring studies have been conducted in South Africa [14], yet billions of Rands have been invested in alien plant clearing. This paper presents the results of topsoil and vegetation monitoring 14 years after *E. grandis* removal by WfW. We used a comparative approach to assess physico-chemical properties in topsoil and native vegetation recovery following the initial *E. grandis* removal in 2008. Our results can provide important information that can be used for the adaptive management of alien plant-cleared areas.

## 2. Results

### 2.1. Effects of Alien Plant Clearing on Soil Properties

The topsoil (hereafter soil) from both the cleared and natural sites were sand (70% and 60%, respectively) and loam (30% and 40%, respectively) soils. Only soil P and Mg were significantly (*p* < 0.01) higher in the natural as compared to the cleared sites (Table 1). Soil P was almost twice higher in the natural as compared to the cleared sites. All other measured soil properties, namely, pH, total C, total N, K, Na, and Ca showed no significant (*p* > 0.05) differences between the cleared and natural sites (Table 1).

**Figure 1:**
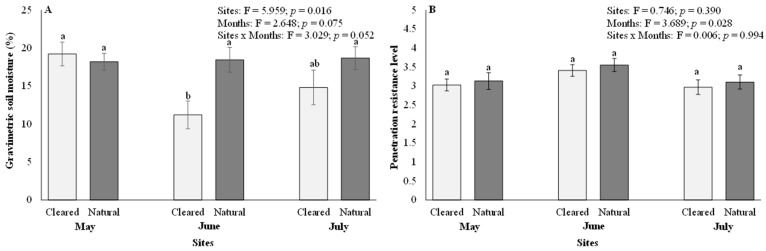
(**A**) Gravimetric soil moisture content, and (**B**) soil penetration resistance levels in cleared and natural sites. Bars represent means ± SE and the ANOVA results are shown. Bars with different letter superscripts are significantly different at *p* < 0.05.

Gravimetric soil moisture content varied significantly between the cleared and natural sites (*p* < 0.05) but not across months (*p* > 0.05; Figure 1A). Significant differences in gravimetric soil moisture content were only visible in June (mean = 11.20 in cleared and 18.45 in natural) but not in May (mean = 19.22 in cleared and 18.20 in natural) and July (mean = 14.81 in cleared and 18.68 in natural) (Figure 1A). The month of June had the lowest gravimetric soil moisture content in the cleared sites (Figure 1A). There were no significant interactions (*p* < 0.05) between the sites and months for gravimetric soil moisture content (Figure 1A). Soil penetration resistance levels showed no significant (*p* > 0.05) differences between cleared and natural sites (Figure 1B). In contrast, monthly comparisons for soil penetration resistance levels showed significant (*p* < 0.05) differences, with July recording the lowest soil penetration resistance levels compared to May and June (Figure 1B). The average soil penetration resistance level across all months was 3.08 in May, 3.48 in June, and 3.04 in July. There were no significant interactions (*p* > 0.05) between the sites and months for soil penetration resistance levels (Figure 1B).

**Figure 2:**
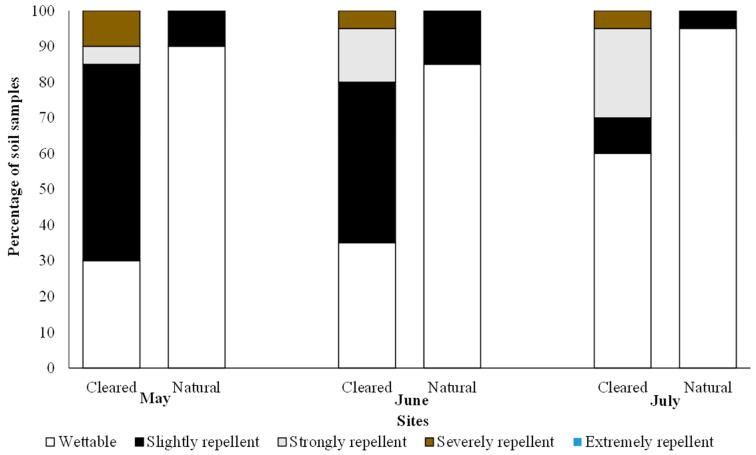
Distribution of the water repellency classes based on the water droplet penetration time method in the soil samples from cleared and natural sites. Chi-squared results are shown.

For soil water repellency, most of the soil in the cleared sites were slightly repellent in May (55%) and June (45%); however, in July the bulk of the soils were wettable (60%) (Figure 2). In the cleared sites, strongly repellent soils were observed across all months, with higher percentages in June (15%) and July (25%) as compared to May (5%). Some of the soil in the cleared sites were severely repellent for all three months (10% in May and 5% in June and July, respectively) as compared to the natural sites, which reported no strongly repellent soils (Figure 2). The bulk of the soils in the natural sites were wettable across all months (May = 90%, June = 85%, and July = 95%) (Figure 2). The remainder of the soils in the natural site were slightly repellent (Figure 2). A chi-squared analysis of the WDPT categories showed significant differences between the cleared and natural sites for all three months (May: χ^2^ = 15.23, *p* = 0.002; June: χ^2^ = 11.17, *p* = 0.011; July: χ^2^ = 7.91, *p* = 0.050).

### 2.2. Effects of Alien Plant Clearing in Vegetation

Although all measured indices of diversity (species richness, Shannon–Wiener, Simpson’s index of diversity, and evenness index) were high in the natural compared to the cleared sites, statistical comparisons showed no significant (*p* > 0.05) differences between the two sites (Table 2). Of all the 50 positively identified plant species, 27 were trees and shrubs, 12 were forbs, and 11 were graminoids and sedges (Table A1). Five plant species, namely, *Asparagus suaveolens*, *Crassula pellucida*, *Helichrysum petiolare*, *Centella asiatica*, and *Conyza bonariensis*, had a frequency occupancy of more than 50% in the cleared sites, while eight species, namely, *A. suaveolens*, *C. pellucida*, *H. cymosum*, *C. asiatica*, *Senecio macrocephalus*, *Agrostis lachnantha*, *Digitaria sanguinalis*, and *Pennisetum clandestinum*, had a frequency occupancy of more than 50% in the natural sites (Table A1). Of all the 27 identified trees and shrubs, 11 were present in both the cleared and natural sites. Half of the identified forbs were in both the cleared and natural sites, and only five graminoids were present in both sites. Two woody invasive alien plants, namely, *A. mearnsii* and *Rubus cuneifolius*, occurred in the cleared sites with a frequency occupancy of less than 25% (Table A1).

## 3. Discussion

Fourteen years after the initial removal of *E. grandis* by WfW, our results show that both the soil physico-chemical properties and the vegetation diversity are recovering in the cleared sites. We observed no significant differences between the cleared and natural sites for most of the measured soil and vegetation variables, an indication that ecosystem recovery is taking place. These results were originally observed by Kerr and Ruwanza [24], who reported a positive vegetation recovery trajectory in one of the cleared sites. However, the same study noted varied clearing effects on the soil properties, both increased and decreased changes. Our results concur with the previous studies that have shown that soil and vegetation recovery tend to follow a positive restoration trajectory several years after the initial clearing [11,14]. Ndou and Ruwanza [14] reported that both soil and vegetation recovery was taking place on old *Acacia*-cleared sites (15 years) than on recently cleared sites (6 years). Similarly, Ruwanza et al. [11] assessed vegetation recovery seven years after *E. camaldulensis* removal along the Berg River and reported a positive vegetation recovery trajectory.

Our results on topsoil showed no significant differences between the cleared and natural sites for all measured soil properties except for P and Mg, an indication that soils in the cleared site resemble those in the natural sites. This contradicts the soil results by Kerr and Ruwanza [24], who observed varied soil nutrient changes. Several factors, including diminishing soil legacy effects after invasive alien plant removal, can explain our soil results [21,23,26]. It is well-documented that soil legacy effects caused by the invader can persist for several years after alien plant removal [21,23,26]; this depends on several factors, such as previous invasion extent, invasion by secondary invaders that add more soil nutrients, and external factors, e.g., grazing and fires, which influence soils [25]. Our results show a possibility that the soil legacy effect reported by Kerr and Ruwanza [24] could be diminishing and is no longer persistent in these cleared sites since the soils are now having similar properties to the ones in natural sites. It is known that the soil legacy effect can limit successful restoration post-alien plant removal [27]; however, in our case, the diminishing soil legacy effect could explain our soil results. Ndou and Ruwanza [14] reported that soil nutrients improve with increased clearing time, with the old cleared sites having similar soil nutrient levels to the natural sites, as compared to the recently cleared sites. In addition to diminishing soil legacy effects, the recovering native vegetation could also explain the observed topsoil nutrient results. It is possible that recruiting native species are using the excessive soil nutrients that were released by *E. grandis* before clearing. The above-mentioned speculation that recovering vegetation is playing a role in soil nutrient changes is plausible, given that similar trends have been observed in abandoned agricultural fields [28]. Studies in abandoned agricultural fields have reported that as woody species colonise grass-dominated abandoned fields, soil nutrients tend to decrease due to increased utilisation by recruiting plants [29]. It is not clear why the soil P and Mg were higher in the natural than in the cleared sites; however, organic matter content from native plant litter could explain this result. Some native species, such as *Maytenus acuminata* and *Pellaea mucronate*, were only present in the natural sites, hence the litter deposition from these species can influence the soil P through soluble P leaching from litter.

We did not observe variations between the cleared and natural sites on soil penetration resistance levels and gravimetric soil moisture content, except for monthly moisture differences, with June recording the lowest soil moisture content. The lack of soil compaction and moisture differences between the two sites could be because of the recruiting vegetation in the cleared sites. Recruiting plant phenological development and increased canopy cover in the cleared sites could have resulted in both soil compaction and moisture being the same as in the vegetated natural sites; however, the seasonal differences in moisture could be because of winter temperature and rainfall patterns. The soil moisture measurements were conducted in the austral winter when rainfall is low; thus, the soils are mostly dry and compact during that time. Several studies have shown that reductions in precipitation tend to lower soil moisture content to as much as 40% during dry months [30], and this reduction in soil moisture also results in increased soil compaction. It was anticipated that the above-mentioned observations in soil nutrients, moisture, and compaction in the cleared sites should have resulted in an improved soil water repellence, but that was not the case, as we recorded a greater percentage of repellent soils in the cleared rather than the natural sites. A palpable explanation is that external factors, such as livestock trampling, which is happening at a low-to-moderate scale in the cleared sites, could explain the reported soil repellence results. The impact of livestock trampling on soil is two-fold, (i) it can trigger soil compaction through decreased soil physical quality and hydraulic conductivity, and (ii) it can result in the detachment and shearing of topsoil layers [31]. However, the effects of livestock trampling on soil repellence remains unknown, with some studies suggesting a reduction in soil water repellence [31], whilst other studies claim an increase in soil water repellency due to increased soil compaction [32].

Our results on vegetation show that native species are recruiting in the cleared sites, an indication that passive native vegetation recovery is taking place. The few long-term studies that have been conducted in South Africa have shown successful native vegetation recovery several years after alien plant removal [11,14]. The above-mentioned studies reported that native species diversity, composition, and cover increase as years since clearing increase [11,14]. Several factors can explain the recruitment of native species in our cleared sites. Firstly, the cleared and natural sites are close to each other; therefore, the natural sites could act as seed suppliers to the cleared sites. The proximity of the cleared sites to the natural sites can assist with native species seed dispersal by animals, such as birds, or through the wind from natural patches to the cleared sites [33]. Secondly, the native recruiting species that were recorded by Kerr and Ruwanza [24] at the same cleared sites could have been established by now, thus acting as nurse plants that facilitate the recruitment of other plants [34,35]. Previous studies have reported that the availability of nurse plants in restoration sites facilitates germination, establishment, and growth of other plant species through (i) attracting birds and insects to disperse seeds underneath them, (ii) providing nutrient-rich microhabitats underneath their canopy that facilitate the germination and growth of other plants, and (iii) buffer recruiting native plants from the harsh environmental conditions [34,35]. Thirdly, it is possible that a native soil seed bank still exists at the cleared sites. Several studies have reported that a soil seed bank of native species can remain in the soil for several years and recruit after the invader has been cleared [36,37]. Fourthly, plant–soil positive interactions could favour the recruitment of native species in the cleared sites. The reported soil nutrient recovery in the cleared sites could benefit recruiting native species through nutrient availability. In turn, recruiting plant species could influence soil properties through litter deposition [38]. Lastly, although livestock grazing can trigger both positive and negative effects on native species recruitment on the cleared sites, it is possible that grazing is assisting with seed dispersal in the cleared sites. Grazing was observed to be more dominant in the cleared sites due to the accessibility by animals since the vegetation is still low and recruiting. Indeed, grazing livestock can disperse native seeds through endozoochory (seed dispersal via ingestion) or epizoochory (seed dispersal accidentally via attachment to animal body) [39,40]. This dispersal has the potential to influence the native species diversity and composition in the cleared sites.

Although Kerr and Ruwanza [24] noted the dominance of secondary invaders and reinvasion by *E. grandis* on our cleared sites, we recorded low abundances of secondary invaders. Even if the observed secondary invasion is diminishing, it still has the potential to slow down native vegetation recovery through competition for resources such as nutrients and water, which alternately hinder native vegetation recovery [11,23]. The observed reinvasion and secondary invasion speak to the challenge of effective follow-up clearing by WfW [11]. As per the South African WfW clearing guidelines, our cleared sites are outside the initial three-year follow-up clearing plan, implying that the property owner is responsible for managing follow-up clearing to remove recruiting *E. grandis* and secondary invaders. However, this could be challenging for the property owner due to a lack of funding, equipment, and human capital to effectively implement follow-up clearing.

## 4. Materials and Methods

### 4.1. Study Area

**Figure 3:**
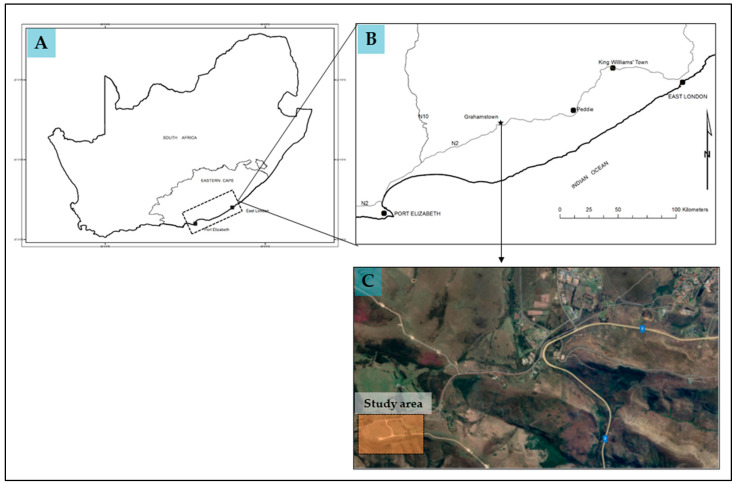
Map showing (**A**) location of study area in South Africa, (**B**) location of study area in the Eastern Cape province of South Africa, and (**C**) farm location (generated using Google Earth Pro Version 7.3 software). National Road (N2) is shown in yellow with highway name in blue.

The study was conducted at a private farm (33°20′24.72″ S; 26°27′11.81″ E) that is approximately 8 km from Makhanda (previously known as Grahamstown; Figure 3) in the Eastern Cape province of South Africa. The farm is currently being used for small-scale livestock grazing. Vegetation in the study areas is dominated by grassy fynbos and small bushveld shrubs [41]. The soils in the area are sandy, acidic, and nutrient-poor, derived from quartzite formation [41]. Rain falls throughout the year with a bimodal distribution, peaking in October-November and February-March [41]. Mean annual rainfall is 545 mm and temperature averages 26 °C in austral summer and 6 °C in austral winter [41].

Within the farm, we identified two cleared and adjacent natural sites. One of the paired cleared and natural sites were surveyed by Kerr and Ruwanza [24], however, it was difficult to identify the exact surveyed plots used in the above-mentioned study since there were removed after the termination of their experiment in 2016. Kerr and Ruwanza [24] assessed similar measurements that were assessed in this study. Our sites were approximately 500 m apart, and the paired cleared and natural areas within each site were separated by farm roads. Clearing of *E. grandis* was performed in 2008 by WfW [24]. Clearing involved the felling of *E. grandis* trees using chainsaws and the spraying of herbicides on cleared stamps to avoid re-sprouting. Felled trees were stack burnt and follow-up treatments to remove re-sprouting alien plants and saplings were conducted on a 4–6 month interval for three years after the initial clearing [24]. After follow-up completion around 2011, the cleared sites were handed over to the property owner for maintenance. Two nearby natural sites acted as reference sites, and these were dominated by native species with a canopy cover of more than 80% [24].

### 4.2. Experimental Design and Data Collection

On each of the paired cleared and natural sites, soil and vegetation surveys were conducted on 10 m × 10 m plots with a buffer zone of 5 m. Each plot was replicated 10 times per site. The plots were marked with metal droppers to allow revisitation during repeated soil measurements. In total 40 plots were surveyed (10 plots per site × 4 sites (2 cleared and 2 natural)). Within each plot, soil cores measuring 10 cm in diameter and 10 cm in depth were collected at the centre of each plot for three months (May to July 2022). Soils were collected using a soil core after hand removal of stones and debris. Collected soils were packed in brown bags and immediately transported to Rhodes University laboratory for gravimetric soil moisture and water repellency measurements. Soil penetration resistance levels were conducted under field conditions, 30 cm from the plot centre where soils were collected. In June, an additional equal number of soils were collected for soil chemical analysis, which was assessed once due to financial limitations and the assumption that no soil chemical variations were expected within one winter season. Soil chemical analyses were conducted at a commercial laboratory, namely, Bemlab (Pty) Limited.

All collected soils were sieved using a 2 mm sieve upon arrival at the laboratory. To measure gravimetric soil moisture, sieved soils were weighed wet, oven-dried at 105 °C for three days and re-weighed to determine moisture content, which was expressed as a percentage [42]. The water droplet penetration time (WDPT) method was used to assess soil water repellency. Sieved soils were placed in Petri dishes, levelled, and air-dried for seven days under laboratory conditions where temperatures averaged 6 °C (±2 °C), similar to winter temperatures in Makhanda. After seven days, the WDPT test was conducted by placing five drops of distilled water on the soil surface using a hypodermic syringe and recording the time taken by each drop to penetrate the soil [24,43]. The average penetration time for the five drops was taken as the WDPT for each sample. The WDPT categories used were wettable (below 5 s), slightly repellent (5–60 s), strongly repellent (60–600 s), severely repellent (600–3600 s), and extremely repellent (above 3600 s) as described by Bisdom et al. [44] and Kerr and Ruwanza [24]. Soil penetration resistance levels (a measure of soil compaction) were performed using a pocket penetrometer (SOILTEST, Inc., Evanston, IL, USA). Measurements were taken in kg cm^−2^ as described by Leung and Meyer [45]. The penetrometer was pushed into the soil following the removal of debris and a metal ring was pushed to scale to record the penetration resistance measurement [45]. Soil pH, a measure of acidity and alkalinity of the soil was analysed in 1:5 soil-KCl extract as described by Rhoades [46]. Soil P was analysed using the Bray-II extract method as described by Bray and Krutz [47]. Soil total C was analysed using the modified Walkley-Black method as described by Chan et al. [48]. Soil total N was analysed by complete combustion using a Eurovector Euro EA Elemental Analyser (Euro EA; Eurovector, Milan, Italy). Exchangeable cations of K, Na, Ca, and Mg were extracted in a 1:10 ammonium acetate solution using the centrifuge procedure described by Thomas [49]. The soils were filtered and analysed using atomic absorption spectrometry (SP428, LECO Corporation, St. Joseph, MI, USA).

In June, detailed vegetation surveys were conducted in each plot. All identified trees and shrubs were counted in each plot, whereas forbs and graminoids were enumerated in a 1 × 1 m sub-plot positioned at the centre of the plot. Species were assigned to four growth forms based on morphology, namely, trees, shrubs, forbs (non-graminoid herbaceous plants), and graminoids [50]. All plant species were identified using local plant books such as Manning [51] and Manning and Goldblatt [52] as well as the PlantzAfrica online directory [53]. Species that could not be identified were taken to Selmar Schonland Herbarium at the Albany Museum in Makhanda for identification.

### 4.3. Data Analysis

All statistical analyses were performed using TIBCO STATISTICA version 14.0 software (TIBCO Software Inc., Palo Alto, California, USA) [54]. Normality tests were performed using the Kolmogorov–Smirnov test and data were normally distributed. The effect of clearing on gravimetric soil moisture and penetration resistance levels was analysed using repeated measures ANOVA since data were collected over three months. Where repeated ANOVAs were significantly different, Tukey’s HSD test was used to determine differences between sites and across months at *p* < 0.05. Comparisons between cleared and natural sites for WDPT categories were performed using the Chi-squared test. Measured soil properties of pH, P, total N, total C, K, Ca, Mg, and Na were compared between cleared and natural sites using a *t*-test since data were collected once. Species richness, Shannon–Wiener diversity index, Simpson’s index of diversity, and Evenness index were calculated per plot and compared between cleared and natural sites using a *t*-test since data were collected once.

## 5. Conclusions and Recommendations

In conclusion, we observed improved soil properties, vegetation diversity, and composition since the last monitoring assessment by Kerr and Ruwanza [24], evidence that ecosystem recovery on these cleared sites is following a positive restoration trajectory towards the natural sites. However, we observed evidence of reinvasion and secondary invasion in low abundance, and this is likely to slow down ecosystem recovery if not attended to. From a management standpoint, some interventions are needed if the current positive recovery trajectory is to be maintained. Although these interventions are yet to be tested and are not prescriptive as they aim to steer restoration conversations, we believe that these interventions need to be considered if clearing by WfW is to yield successful native vegetation recovery. Firstly, long-term restoration monitoring should be included in alien plant clearing and management plans, and such monitoring should be performed until restoration is completely achieved. Secondly, clearing managers should develop an effective and timeous follow-up clearing programme that monitors reinvasion and secondary invasion on cleared sites. Thirdly, there is a need to support landowners with resources to manage the cleared sites post the initial WfW follow-up period. Support to landowners could be in the form of financial resources to buy follow-up clearing chemicals, pay human capital, and information on how to manage the cleared sites. Lastly, future research on cleared sites should assess both topsoil and below-ground soil properties to provide accurate and detailed information on recovery after alien plant removal.

## Figures and Tables

**Table 1:** Comparison of soil physical and chemical attributes between cleared and natural sites. Data are means ± SE and *t*-test results are shown.

	Cleared	Natural	*t*-Values	*p*-Values
Soil pH	4.43 ± 0.19	4.11 ± 0.04	1.66	0.115
Total nutrient concentrations				
P Bray II (mg/kg)	3.66 ± 0.47	7.87 ± 1.27	3.11	0.006
C (%)	2.90 ± 0.25	3.51 ± 0.32	1.50	0.151
N (%)	0.23 ± 0.02	0.30 ± 0.04	1.50	0.150
Exchangeable cations (%)				
K	6.22 ± 1.63	8.77 ± 2.50	0.85	0.404
Na	2.49 ± 0.19	2.80 ± 0.23	1.02	0.323
Ca	38.43 ± 1.20	30.28 ± 4.31	1.82	0.085
Mg	14.46 ±1.65	20.19 ± 0.40	3.38	0.003

**Table 2:** Comparison of indices of diversity between the cleared and natural sites. Data are means ± SE and *t*-test results are shown.

	Cleared	Natural	*t*-Values	*p*-Values
Species richness	8.65 ± 0.51	10.25 ± 0.63	1.96	0.057
Shannon–Wiener	1.25 ± 0.06	1.40 ± 0.08	1.51	0.139
Simpsons index of diversity	0.72 ± 0.04	0.74 ± 0.02	0.69	0.495
Evenness index	0.59 ± 0.03	0.61 ± 0.02	0.68	0.679

## Data Availability

The data that support our research findings are available from the corresponding author on request.

## Appendix A

**Table A1:** Fifty frequently occurring species in cleared and natural sites.

Species Name	Cleared	Natural
*Acacia mearnsii*	25	0
*Anthospermum aethiopicum*	5	0
*Anthospermum spathulatum*	30	15
*Aspalathus subtingens*	0	35
*Asparagus suaveolens*	50	80
*Athanasia dentata*	5	15
*Chasmanthe aethiopica*	0	5
*Clutia daphnoides*	0	5
*Conyza scabrida*	0	5
*Crassula pellucida*	50	60
*Dovyalis rhamnoides*	5	5
*Eucalyptus grandis*	30	0
*Halleria lucida*	5	15
*Helichrysum cymosum*	20	70
*Helichrysum petiolare*	65	45
*Indigofera* sp.	5	10
*Maytenus acuminata*	0	5
*Metalasia muricata*	5	20
*Passerina rigida*	0	15
*Podocarpus latifolius*	0	5
*Protea neriifolia*	0	10
*Psychotria capensis*	0	5
*Pteronia incana*	0	25
*Searsia crenata*	25	15
*Rubus cuneifolius*	5	0
*Solanum linnaeanum*	10	0
*Zanthoxylum capense*	0	5
*Asplenium rutifolium*	5	20
*Centaurea benedicta*	15	0
*Centella asiatica*	70	65
*Conyza bonariensis*	95	20
*Erigeron canadensis*	5	0
*Erigeron* sp.	10	10
*Euphorbia epicyparissias*	0	15
*Pellaea mucronate*	0	25
*Pteridium aquilinum*	0	10
*Senecio macrocephalus*	40	60
*Thesium gnidiodes*	45	40
*Wahlenbergia procumbens*	5	0
*Agrostis lachnantha*	30	55
*Alloteropsis semialata*	25	0
*Aristida transvaalensis*	5	0
*Carex* sp.	20	20
*Cynodon dactylon*	5	0
*Cyperus albostriatus*	20	0
*Digitaria sanguinalis*	25	65
*Ehrharta erecta*	35	0
*Isolepis cernua*	5	0
*Pennisetum clandestinum*	20	50
*Sporobolus africanus*	20	5
